# Interneuron Dysfunction and Inhibitory Deficits in Autism and Fragile X Syndrome

**DOI:** 10.3390/cells10102610

**Published:** 2021-10-01

**Authors:** Toshihiro Nomura

**Affiliations:** Department of Neuroscience, Feinberg School of Medicine, Northwestern University, Chicago, IL 60611, USA; toshihiro.nomura@northwestern.edu

**Keywords:** autism, fragile X syndrome, interneuron, GABA, E–I balance

## Abstract

The alteration of excitatory–inhibitory (E–I) balance has been implicated in various neurological and psychiatric diseases, including autism spectrum disorder (ASD). Fragile X syndrome (FXS) is a single-gene disorder that is the most common known cause of ASD. Understanding the molecular and physiological features of FXS is thought to enhance our knowledge of the pathophysiology of ASD. Accumulated evidence implicates deficits in the inhibitory circuits in FXS that tips E–I balance toward excitation. Deficits in interneurons, the main source of an inhibitory neurotransmitter, gamma-aminobutyric acid (GABA), have been reported in FXS, including a reduced number of cells, reduction in intrinsic cellular excitability, or weaker synaptic connectivity. Manipulating the interneuron activity ameliorated the symptoms in the FXS mouse model, which makes it reasonable to conceptualize FXS as an interneuronopathy. While it is still poorly understood how the developmental profiles of the inhibitory circuit go awry in FXS, recent works have uncovered several developmental alterations in the functional properties of interneurons. Correcting disrupted E–I balance by potentiating the inhibitory circuit by targeting interneurons may have a therapeutic potential in FXS. I will review the recent evidence about the inhibitory alterations and interneuron dysfunction in ASD and FXS and will discuss the future directions of this field.

## 1. Introduction

This review will discuss the pathophysiology of autism and fragile X syndrome (FXS) from a neurophysiological viewpoint. I will take FXS as a model to understand autism pathophysiology and will put this autism-related disorder at the center of the discussion. Balanced excitation and inhibition in the brain is critical to maintaining proper neuronal function. Alterations both in excitation and inhibition have been described in autism and FXS, but the inhibitory system will be the main focus in this review. I will particularly emphasize interneuron dysfunction as an important component responsible for inhibitory alterations. Taking the developmental characteristics of autism and FXS into account, how the developmental profile of the inhibitory system goes awry will also be precisely described. I will summarize and discuss the published evidence and will present future perspectives, particularly from a translational aspect, in this field.

## 2. Excitatory–Inhibitory (E–I) Imbalance

It is now cliché to say “Excitatory–Inhibitory (E–I) balance is disrupted in XXX disorders”. Intuitively, disrupted E–I balance has been rigorously studied in paroxysmal disorders where “more excitatory” and/or “less inhibitory” brain activity is associated with epileptic seizures. While this straightforward connection makes sense, the hypothesis that E–I imbalance is causal to a multitude of behavioral symptoms is undoubtedly too simplistic [1,2,3]. “XXX disorders” now, however, range from paroxysmal disorders to various other disorders, including autism [2,3,4,5,6]. Autism and epilepsy are commonly comorbid conditions, and the genesis for the altered E–I balance theory in autism was because of this co-occurrence [5].

In 2003, Rubenstein and Merzenich published a seminal review in which they proposed a model in which E–I balance was shifted towards excitation that was the basis for the pathological brain circuitry function in autism [7]. Alterations both in the excitatory and inhibitory systems can result in E–I imbalance. Multiple factors, including the number of neurons, intrinsic excitability of neurons, synaptic connectivity, and network activity, can all be critical components regulating E–I balance [3]. Indeed, both excitatory and inhibitory alterations have been reported in autism [8]. In this review, inhibitory alterations, particularly dysfunction of interneurons, the main source of an inhibitory neurotransmitter gamma-aminobutyric acid (GABA), will be the primary focus.

## 3. Autism and Fragile X Syndrome

Autism is a neurodevelopmental disorder that is conceptualized as a spectrum of disorders (autism spectrum disorder; ASD). ASD individuals have difficulties in social behaviors and communication skills as core symptoms, but comorbid symptoms such as stereotyped movements, restricted interests, anxiety, or sensory hypersensitivity vary considerably in the type and magnitude among individuals [9,10,11]. The heterogeneity of ASD, which likely involves multiple genetic and environmental factors, makes it challenging to clarify the pathophysiological mechanism [12,13]. Several single-gene or chromosomal disorders—for instance, FXS—have a high incidence of co-occurrence of ASD-like symptoms, and it is expected that understanding these disorders may lead to elucidating the pathophysiology for the development of new therapeutic interventions for ASD [10,14].

FXS is the most common (with a prevalence of 1 in 4000–5000 in males and 1 in 6000–8000 in females, but it varies considerably among studies [15,16]) known cause of intellectual disability and ASD [17,18]. FXS results from an epigenetic silencing of the responsible gene *FMR1* caused by an abnormal expansion of a CGG repeat sequence in the 5′ untranslated region, leading to hypermethylation, transcriptional silencing, and a loss of expression of the protein product fragile X mental retardation protein (FMRP) [19,20,21]. FMRP is an RNA-binding protein enriched in neurons, epithelial tissues, and testes that regulates the expression of other genes as a translational repressor [22,23,24]. Animal models are particularly useful tools to understand the molecular, cellular, and circuit phenotypes, because they allow an accessibility to study neuronal and circuit functions, which is very difficult or practically impossible in affected human subjects. In FXS, multiple animal models are available and commonly used, including *Fmr1* knock-out (KO) mice [25], *Dfxr* (Drosophila fragile X-related gene) null drosophila [26], *Fmr1* KO zebrafish [27], and *Fmr1* KO rats [28]. The “knock-in” of the expanded CGG repeat did not work well in mice to model FXS phenotypes [29,30,31].

As a pathological phenotype, disruption in E–I balance has been reported in FXS. The target of FMRP includes RNAs encoding synaptic proteins and trophic factors [21,32,33,34,35]. Therefore, it is not surprising that multiple morphological and functional alterations affecting E–I balance exist in FXS. Although it is not the central focus of this review, the excitatory neurotransmitter system has been intensively studied in FXS for more than two decades, and studies have revealed that glutamatergic transmission goes awry in FXS [36,37,38].

## 4. Inhibitory System Alteration

The inhibitory neurotransmitter system is a counter component to the excitatory system determining E–I balance. There are multiple lines of evidence for inhibitory alterations in individuals with FXS and in the animal models [21,39]. Circuit hyperexcitability is consistently reported in FXS. Though there is huge variability in the prevalence among the cohorts, FXS individuals have a high susceptibility to seizures, electroencephalogram (EEG) abnormalities, and epilepsy syndromes [14,39,40], which may be, in part, due to an abnormal inhibitory system. GABA is the main source of inhibitory transmission and may play an important role in dysfunction in the inhibitory system in FXS [41,42], but direct evidence for a dysfunctional GABA system in human subjects is relatively limited. A study using positron emission tomography (PET) in a small cohort of patients showed a significant reduction of approximately 10% in GABA_A_ receptor availability throughout the brain, which may cause diminished GABA-mediated inhibition in FXS individuals [43]. A recent finding using transcranial magnetic stimulation (TMS) demonstrated that FXS patients had significantly increased intracortical facilitation (ICF), reduced short-interval intracortical inhibition (SICI), and increased long-interval intracortical inhibition (LICI), which are believed to represent glutamatergic excitation, GABA_A_-mediated inhibition, and GABA_B_-mediated inhibition, suggesting that cortical hyperexcitability is, in part, due to reduced GABA_A_ inhibition in FXS individuals [44].

In FXS animal models, however, aberrant GABA signaling has been reported in multiple studies. At the behavioral level, there is evidence indicative of the altered inhibition. *Fmr1* KO mice have a lower threshold for audiogenic seizures, which is one of the most robust and reliably reported behavioral phenotypes in this mouse line [45,46]. Audiogenic seizures are possibly triggered by neuronal and circuit hyperexcitability due to altered sensory processing and sensory hypersensitivity in the auditory system [21,45,47]. Moreover a causal relationship between diminished GABA-mediated inhibition and audiogenic seizures has been established, as agonists of GABA_A_ [48,49] and GABA_B_ [50,51] receptors can rescue enhanced audiogenic seizures in *Fmr1* KO mice, and antagonists of GABA_B_ receptors mimic the *Fmr1* KO phenotype [50].

Pre-pulse inhibition (PPI) is another indicator for auditory hypersensitivity, and multiple studies have reported increased PPI in *Fmr1* KO mice [52]. Increased PPI in mouse models is contradictory to the finding in FXS individuals where PPI is reported to be decreased [53]. A study using eye blink responses [54] and a recent study in young (P23–25) *Fmr1* KO mice [55] have shown a significantly reduced PPI, which is somewhat reminiscent of the findings in human FXS individuals. Importantly GABA_A_ receptor activation by its agonist 4,5,6,7-tetrahydroisoxazolo(5,4-c)pyridin-3-ol (THIP) ameliorates increased PPI in *Fmr1* KO mice, suggesting a causal relationship between decreased GABA inhibition and altered PPI [52]. Alterations in PPI may also, in part, arise from sensory hypersensitivity to somatosensory stimuli, which is also one of the most commonly shared phenotypes in FXS individuals. Evidence shows a negative correlation in the GABA concentration in the somatosensory cortex to tactile sensitivity in humans [56,57,58,59]. In animal models of FXS, a recent study demonstrated that sensory hypersensitivity can be reliably monitored even in young (<P14) and in adult *Fmr1* KO mice, which is associated with a deficit in an adaptation in cortical neuronal response activity in the somatosensory cortex to repetitive tactile stimuli [60].

## 5. ASD and FXS Are Interneuronopathy

The concept of “interneuronopathy” was originally proposed in X-linked lissencephaly with abnormal genitalia (XLAG) [61], in which deficient tangential migration and a loss of inhibitory interneurons were described [61,62]. It was later expanded to other intractable epilepsy syndromes, such as Dravet syndrome [63,64] or West syndrome [65,66], in which deficits in the development, the number, and the function of interneurons are thought to contribute to clinical manifestations such as epileptic seizures. Interneurons have local axons and innervate adjacent principal projection neurons and other interneurons [67,68]. Interneurons are composed of diverse subpopulations of neurons characterized by the expression of specific marker proteins such as parvalbumin (PV), somatostatin (SST), or vasoactive intestinal peptide (VIP) [67,68] (but there are also multiple different ways to determine interneuron subclasses, including recently proposed ways based on single-cell transcriptome or connectivity and projection pattern: please see these articles about interneuron diversity for more details [68,69,70,71,72,73]). While interneurons represent a relatively minor population (~20%) of all neurons in the brain [67,74,75], they are a source of GABA and serve as a main source of inhibitory transmission, suggesting their indispensable roles in physiological and pathological conditions.

**Figure 1:**
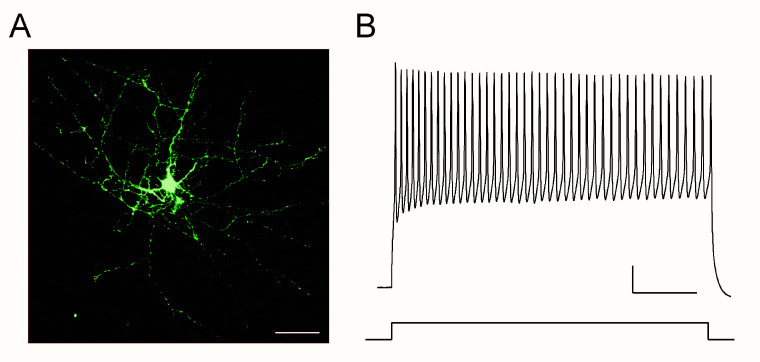
(**A**) PV-positive cortical interneuron filled with biocytin labeled with streptavidin-conjugated AlexaFluor 488. Alterations in this subclass of interneurons are consistently reported in ASD. Scale bar: 50 μm. (**B**). Typical firing patter of PV interneurons in response to depolarizing current injection (100 pA for 500 ms). These neurons fire action potentials at a high frequency constantly for a prolonged period. The inter-spike intervals do not largely change during the prolonged spike train, i.e., fast-spiking. Calibration: 100 ms and 25 mV (from Reference [81] with edits).

There is now growing evidence about interneuron dysfunction in ASD and FXS. While it is not conclusive whether interneuron dysfunction is the primary pathological mechanism, it motivates researchers to consider these disorders to be interneuronopathies [8,76,77]. In human research, postmortem studies have consistently reported reduced cell density of PV positive fast-spiking interneurons, one of the most major classes of interneurons [67] (Figure 1), in ASD individuals [77]. In FXS individuals, however, direct evidence is limited about interneuron phenotypes. The deficit in interneurons in FXS was originally reported in the mouse model. Selby et al. first described a remarkable (~20%) decrease in the cell density of PV interneurons in the somatosensory cortex in *Fmr1* KO mice [78]. Similar findings were reported in the developing auditory cortex, and perineuronal net (PNN) formation, which is the extracellular matrix associated with PV interneurons, is also impaired in the same mouse line [79]. These findings are interesting given the TMS study in human subjects showing a reduced SICI in FXS [44], because TMS is believed to act predominantly through modulating PV interneurons [80].

Not only are the reductions in the number of cells, but also, the functional alterations in inhibitory interneurons, particularly in PV interneurons, are implicated in multiple studies in FXS animal models [77]. Early works demonstrated that, in the amygdala, there is a drastic reduction both in phasic and tonic inhibitory transmission in *Fmr1* KO mice [82]. This reduced inhibition is likely caused by functional deficits in presynaptic neurons in inhibitory synaptic circuits, i.e., GABAergic interneurons, as the GABA synthesizing enzymes glutamate decarboxylase (GAD) 65/67 levels and synaptic GABA availability are reduced in *Fmr1* KO mice [82]. In vivo recording results in sensory cortices agree with the reduced interneuron activity in *Fmr1* KO mice [83] and *Fmr1* KO rats [84]. Impaired interneuron function was also implicated in a drosophila model of FXS [85]. The optogenetic activation of interneurons fails to elicit as strong lateral inhibition in *Dfmr1* KO flies as in wild-type (WT), which indirectly indicates impaired interneuron function and outputs [85]. Probably one of the most direct and important pieces of evidence about abnormal interneuron function is a recent finding in the visual cortex in *Fmr1* KO mice [86]. The activity of PV interneurons was directly monitored using in vivo Ca^2+^ imaging technique during a visual discrimination task [86]. *Fmr1* KO mice showed poor behavioral performances in the task, and the interneuron activity was reduced in *Fmr1* KO mice [86]. Most importantly, artificial activation of these neurons by designer receptors exclusively activated by designer drugs (DREADD) restored the impairment in the behavioral performance, which strongly suggests a causal relationship between the neuronal activity and the behavioral phenotype [86]. In addition, the poor performance in the behavioral task was fully translated in human FXS individuals [86]. Evidence for alterations in other subclasses of interneurons such as SST or VIP-positive interneurons is limited in ASD and FXS.

Although multiple studies have suggested reduced inhibition due to reduced interneuron activity in animal models of FXS, evidence in the cerebellum of *Fmr1* KO mice counteracts that idea [87]. Cerebellar basket cells (BCs), a major class of cerebellar interneurons, exhibit hyperactivity in their axonal terminals, leading to an exaggerated GABAergic inhibition onto the principal neuron Purkinje cells (PCs) and reduced firing of PCs in *Fmr1* KO mice [87]. This hyper-inhibition is caused by a loss of direct modulation of the voltage-gated potassium channel Kv1.2 by the N-terminal of FMRP [87]. Given the ubiquitous expression of Kv1.2 across the brain, the mechanism for this dichotomy (increased inhibition in the cerebellum vs. reduced inhibition in other brain regions) cannot be easily explained. Interestingly, exaggerated inhibitory transmission and/or the reduced firing of PCs have been reported in multiple ASD animal models, including in *Tsc1* [88], *PTEN* [89], *Shank2* [90] mutants, and BTBR [91] mouse lines. Therefore, it might be possible that these cerebellar cellular and circuitry phenotypes represent certain aspects of ASD. Given the fact that the cerebellum is implicated to be one of the key brain regions responsible for the pathophysiology of ASD [92], further studies are clearly needed to explore this regional specificity or discrepancy with other brain regions.

## 6. Developmental Alteration

Given the characteristics of FXS as a developmental disorder, understanding the developmental profiles of the brain circuit is undoubtedly indispensable. However, current evidence about the circuit development is relatively limited compared to the large number of studies in adult FXS animal models. Here, I will focus on evidence in the somatosensory cortex in which several studies have uncovered the abnormal development of both excitatory and inhibitory circuits. Several studies have demonstrated transient alterations in the morphology and turnover of dendritic spines [93,94,95,96,97,98], functional maturation of excitatory synapses [99,100], and the excitatory innervation pattern [101] during the somatosensory critical period (1 to 2 postnatal weeks) in *Fmr1* KO mice. The closure of the critical period for synaptic plasticity was delayed in the somatosensory cortex in *Fmr1* KO mice [99,100]. These series’ of developmental studies have demonstrated that alterations in the excitatory synapses are parallel with the time course of the sensory critical period. Importantly, the timing of the critical period closure is largely determined by cortical inhibition, particularly by PV interneurons, which have been rigorously studied in the visual cortex [102].

**Figure 2:**
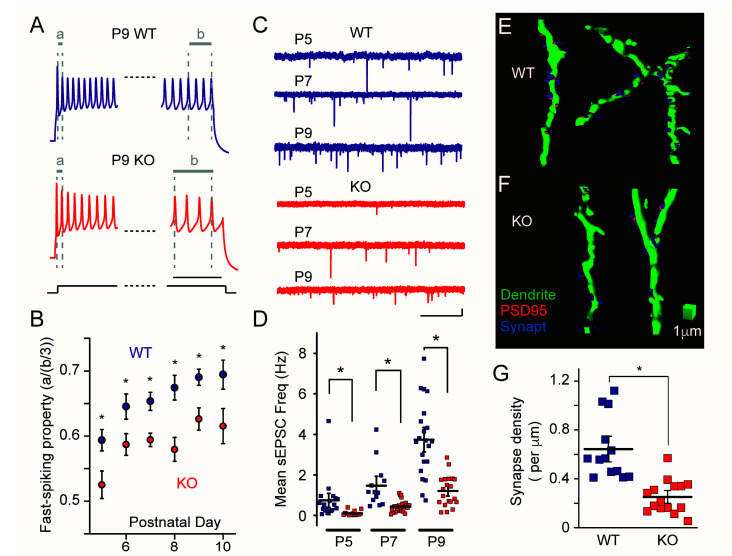
(**A**) Representative voltage response traces of PV interneurons in developing (postnatal day 9: P9) *Fmr1* WT (top) and KO (bottom) mice. The initial and final portions of the 500-ms-long traces were magnified for clarity. Note the significantly greater ratio of the last three inter-spike intervals “b” to the first inter-spike interval “a” in KO mice compared to in WT mice, which indicates that PV interneurons equip less fast-spiking properties in KO mice. Calibration: 50 ms. (**B**) Collective data for fast-spiking property during the development (P5–P10) in *Fmr1* WT and KO mice. The spiking property was quantified by “a” divided by “b/3”. * Denotes *p* < 0.05. (**C**) Representative sEPSC traces recorded from developing (P5–P9) PV interneurons in *Fmr1* WT (top) and KO (bottom) mice. Calibration: 1 s and 10 pA. (**D**) Collective data for sEPSC frequency. Each data point represents the mean sEPSC frequency in each cell. The frequency increases developmentally but remains lower in *Fmr1* KO mice. (**E**,**F**). Three-dimensional renderings of dendritic segments (green) of PV interneurons in *Fmr1* WT (**E**) and KO (**F**) mice. Synapses are labeled with the colocalized puncta of postsynaptic marker PSD95 (red) and presynaptic marker synaptophysin (“Synapt”: blue). The reference cube represents 1-μm calibrations. (**G**) Collective data for the analysis of the synapse densities in *Fmr1* WT and KO mice (P9–P10). The density of the synapses was significantly lower in *Fmr1* KO mice (from Reference [81] with edits).

Studies have uncovered developmental cellular and synaptic alterations in PV interneurons during the sensory critical period. The intrinsic membrane properties and characteristic fast-spiking firing patterns exhibit an immature profile in the developing (P5–P10) somatosensory cortex (layer 4) in *Fmr1* KO mice [81] (Figure 2A,B). Another study demonstrated that PV interneurons show immature passive membrane properties but fire an action potential more easily (lower rheobase current) in P10 *Fmr1* KO mice [103]. These studies demonstrated that the functional maturation of PV interneurons is significantly delayed in *Fmr1* KO mice. The development of excitatory synapses onto PV interneurons is also significantly delayed during the critical period. The frequency of the spontaneous excitatory postsynaptic current (sEPSC) recorded from PV interneurons is lower, and the density of the synapses was decreased in <P10 *Fmr1* KO mice, which suggests that the bulk excitatory synaptic input and the number of synapses are reduced in PV interneurons in *Fmr1* KO mice [81] (Figure 2C–G). The synaptic connection probability and the strength of the individual synapses between excitatory stellate neurons, the principal neurons in layer 4, and PV positive fast-spiking interneurons are also reduced in 2-week-old *Fmr1* KO mice, which again suggests that the excitatory synaptic inputs onto PV interneurons are weaker in *Fmr1* KO mice [103,104,105]. These findings indicate that PV interneurons demonstrate immature and defective activity both intrinsically and synaptically during the critical period in *Fmr1* KO mice.

The deficits in the intrinsic and synaptic properties in PV interneurons result in antagonistic complex alterations in thalamocortical circuit activity during the critical period. Thalamocortical feed-forward inhibition (FFI) involves a di-synaptic circuit in which inhibition is mediated by PV interneurons [106,107]. The FFI is deficient (lacked in a subset of cells) in the somatosensory cortex (layer 4) in P10 *Fmr1* KO mice [103] Nomura et al. unpublished. Thalamic excitatory afferents produce action potentials in stellate cells more readily but with less precise timing upon stimuli at physiologically relevant frequencies as a result of deficiency in FFI [103]. While some cellular and synaptic measures of PV interneurons are normalized as the mice develop [81,104,105], as with excitatory synapses [94,99,101], there are persistent circuit-level (including FFI in layer 2/3 [83]) and behavioral-level phenotypes in adult *Fmr1* KO mice beyond the sensory critical period [60,83,100,108,109,110]. This indicates that transient cellular and synaptic deficits in PV interneurons during the critical period can still result in persistent improper thalamocortical circuit refinement, which might warrant correcting these alterations during the developmental period is critical to treat symptoms in *Fmr1* KO mice. Indeed, manipulating GABA signaling by a Cl^−^ transporter modulator, which alters the GABA receptor polarity during the critical period, restores the abnormal whisker-evoked responses (wider receptive field and stronger signal) in the adult somatosensory cortex in *Fmr1* KO mice [100]. Whether manipulating the interneuron activity during the critical period has persistent beneficial effects in *Fmr1* KO mice has not been tested, but several approaches, including optogenetic or chemogenetic tools, to specifically target interneurons may help testing this critical period-interneuron hypothesis.

In addition to animal models, using human pluripotent cells such as induced pluripotent stem cells (iPS) or embryonic stem (ES) cells allows analyses of developmental profiles in vitro, which is not easy or even impossible in embryos of human patients. This approach is also useful for neurodevelopmental disorders, including ASD and FXS [111,112]. Evidence specific to interneurons in human-derived neurons is limited, but functional analyses using human ES cell-derived excitatory neurons from FXS individuals uncovered impaired action potential firing, which may be, in part, due to reduced activity in voltage-gated Na^+^ and K^+^ channels, as well as reduced synaptic excitation, which may be attributable to the reduced excitability and impaired vesicular release in the presynaptic neurons [113,114]. Similar approaches could be applied to study the interneuron pathology in human FXS, particularly developmental alterations in combination with experimental protocols, to specifically differentiate interneurons from pluripotent cells [115,116,117,118].

## 7. Translational Perspective

As discussed, there is now considerable evidence to believe the inhibitory deficits and interneuron dysfunction in the pathophysiology of FXS and ASD. It is theoretical and justifiable to consider the GABAergic inhibitory system as a promising therapeutic target for FXS [39,42]. However, up to now, there have been no successes in clinical trials in FXS that have tested GABA-targeting drugs. Arbaclofen, which is an agonist of GABA_B_ receptors, was not effective in FXS individuals in phase 2 and phase 3 clinical trials, while the post hoc analyses showed several beneficial results in several measures, which might suggest younger and more severely affected individuals may have some beneficial effects [119,120]. Ganaxolone, which is an agonist of δ subunit-containing GABA_A_ receptors, was not effective in FXS individuals in a phase 2 clinical trial, while the post hoc analyses presented some potential where individuals with higher anxiety or lower cognitive function may have more beneficial effects [121]. These clinical studies may have indicated these treatments might be effective in a subpopulation of affected individuals, depending on age, specific symptoms, or severity, for instance. However, it appears not to be game-changing.

It might be worth noting that most clinical research so far has targeted GABA receptors, the postsynaptic component of the inhibitory circuit. Given evidence is accumulating about the dysfunction of inhibitory interneurons, the presynaptic component of the inhibitory system, targeting these presynaptic neurons might be worth considering. Though manipulating a certain population of neurons is challenging, particularly in live human brains, cell type-specific proteins such as channels, receptors, or transporters specifically expressed in certain synapses or in certain circuits might be potential candidates to be considered. For instance, Kv3.1, a subfamily of voltage-gated potassium channels, is enriched in high-frequency firing neurons such as PV-positive fast-spiking GABAergic interneurons, glycinergic medial nucleus of the trapezoid body (MNTB) neurons, and cerebellar Purkinje neurons [122]. Modulators of Kv3.1 have been shown to manipulate neuronal excitability in PV interneurons [123,124] and MNTB neurons [125]. A recent study demonstrated that AUT2, a positive modulator of Kv3.1, restored an enhanced wave IV in the auditory brainstem response (ABR) recordings in *Fmr1* KO mice, which may have suggested a therapeutic potential for sensory symptoms in FXS individuals, although the pharmacological actions for ABR rescue are likely through modulating the activity of MNTB neurons, not GABAergic interneurons [126]. Further studies for the cell type and circuit-specific analyses might be helpful for developing novel therapeutics in the future.

While the GABAergic inhibitory system looks like a reasonable therapeutic target for ASD and FXS [39,42], recent research has critically questioned if E–I imbalance particularly reduced inhibition is causal for ASD pathophysiology [83]. It has been demonstrated that multiple ASD mouse models, including *Fmr1* KO mice, show E–I imbalance towards excitation and reduced interneuron activity in the somatosensory cortex (layer 2/3), but the network excitability (postsynaptic depolarization and sensory-evoked firing) is largely unchanged or even reduced in *Fmr1* KO mice [83]. The authors concluded that altered E–I balance is compensatory, instead of causal, change to stabilize depolarization and spiking [83]. If this compensatory model is the case, targeting the inhibitory system to enhance the inhibition by GABA receptor activators, for instance, may amplify, instead of ameliorate, the symptoms of ASD and FXS. In addition, recent studies have shown that the frequency of basal spontaneous inhibitory postsynaptic current (sIPSC) recorded from principal neurons in the somatosensory cortex (layers 2/3) [127] and in the lateral amygdala [128] is enhanced, which may reflect the circuit hyperexcitability in *Fmr1* KO mice. Synaptic plasticity in excitatory and inhibitory synapses are exaggerated and diminished, respectively, in *Fmr1* KO mice [127,128], which might help to stabilize the homeostatic circuit activity. These findings may again question if correcting synaptic GABA signaling is beneficial for FXS. Further studies are clearly needed to clarify and tackle this issue.

More generally, not restricted to ASD or FXS, successful translational research, particularly in seeking new therapeutics, is extremely rare. It is known that more than 80% of the candidate compounds that demonstrated safety and efficacy in preclinical studies have no beneficial effect in human patients [129]. This discrepancy may arise from multiple complex factors, including the heterogeneity of the disorder, a lack of objective biomarkers and measures of the outcome, invalid sample size, and publication and citation biases towards positive results [129,130,131,132,133,134,135]. Registered reports in which research proposals are registered and reviewed before experiments are started and publication is guaranteed regardless of the outcome (whether positive or negative/novel or not novel) may reduce some of these biases and may have a potential to serve as an alternative platform in translational research, particularly in hypothesis-driven studies [136,137].

## 8. Conclusions

Decades of research uncovered circuit E–I imbalance as a shared feature in various brain disorders, including ASD. Single-gene disorders, including FXS, have been serving as a good model to study ASD pathophysiology together with their animal models. Evidence is accumulating about alterations in the inhibitory system, and it looks like a promising target to seek new therapeutics in ASD and FXS. Interneuron dysfunction is an important contributor to impaired inhibition, and it may also be causal for the symptoms of ASD and FXS. Evidence is accumulating about developmental alterations, but we are at an early stage of understanding. The pharmacological and modern chemogenetic manipulations of this inhibitory system ameliorate the pathological symptoms in animal models, but no successful clinical trials targeting GABA receptors are available so far. The successful translation from basic research to clinical practice is rare, which should not be overlooked. Although we are still far behind a complete picture for the satisfactory understanding of ASD and FXS, intense collaborative studies between clinical and basic researchers may promote our knowledge about these intractable disorders.

## Data Availability

Not applicable.
